# Decoding the Neurological Connection Between Rosacea and Migraines: Exploring Shared Mechanisms

**DOI:** 10.7759/cureus.108668

**Published:** 2026-05-11

**Authors:** Aymen Arain, Maryam Babar, Nina Martins, Danny Lee

**Affiliations:** 1 Department of Medicine, Edward Via College of Osteopathic Medicine, Monroe, USA; 2 Department of Dermatology, The University of Texas Medical Branch, Galveston, USA; 3 Department of Internal Medicine - Preliminary Program, The University of Tennessee Health Science Center, Memphis, USA; 4 Department of Internal Medicine, University of California San Francisco, San Francisco, USA

**Keywords:** calcitonin gene-related peptide (cgrp), facial flushing, migraine, neurogenic inflammation, neurovascular disorders, pituitary adenylate cyclase-activating polypeptide (pacap), rosacea, sensory hypersensitivity, transient receptor potential channels (trp channels), trigeminovascular system

## Abstract

Rosacea and migraine are prevalent chronic disorders traditionally viewed as distinct conditions affecting separate organ systems. However, emerging evidence suggests a significant overlap, notably through shared neurovascular and neuroinflammatory pathways. This literature review explores the mechanisms linking rosacea, a chronic inflammatory skin condition characterized by facial flushing and sensitivity, and migraine, a neurological disorder marked by recurrent headaches. Current research highlights the critical roles of neuropeptides, including calcitonin gene-related peptide (CGRP), substance P (SP), and pituitary adenylate cyclase-activating polypeptide (PACAP), in mediating inflammation and vascular dysregulation common to both conditions. Despite these advances, notable gaps remain, such as limited data on the impact of migraine treatments (e.g., CGRP inhibitors) on rosacea, unclear reasons behind the selective comorbidity of these conditions, minimal research comparing rosacea to other headache disorders, and methodological limitations across studies. Addressing these gaps through interdisciplinary research holds promise for improved clinical outcomes. This review underscores the importance of recognizing rosacea and migraine as interrelated neurovascular disorders, advocating for integrated therapeutic approaches, and proposing directions for future research.

## Introduction and background

Rosacea is a chronic inflammatory skin disorder characterized by recurrent facial flushing, persistent erythema, papules, pustules, phymatous changes, telangiectasias, and stinging or burning sensations [1]. With a global prevalence estimated at approximately 5-6% of adults, rosacea is a common yet often underdiagnosed condition that has traditionally been viewed as a primarily cutaneous disease [1,2]. However, accumulating evidence links rosacea with a range of systemic comorbidities, including neurologic, cardiovascular, and gastrointestinal disorders, suggesting that it may represent a broader systemic inflammatory phenotype rather than an isolated skin disorder [1]. Rosacea is currently classified into several clinical phenotypes, including erythematotelangiectatic rosacea, papulopustular rosacea, phymatous rosacea, and ocular rosacea, with many patients demonstrating overlapping features rather than fitting neatly into a single category [1].

Migraine is a highly prevalent primary headache disorder characterized by recurrent attacks of moderate-to-severe headache, typically unilateral and throbbing, and often accompanied by nausea, photophobia, and phonophobia [3]. Contemporary global burden analyses indicate that migraine affects roughly 14-15% of the world’s population and ranks among the leading causes of years lived with disability, particularly in young and middle-aged adults [3,4]. Both rosacea and migraine are therefore chronic, relapsing conditions with substantial impact on quality of life, work productivity, and healthcare utilization [1,3,4].

Clinical and epidemiologic observations increasingly suggest a non‑random association between rosacea and migraine [5,6]. Many patients with either condition report recurrent symptoms localized to the trigeminal distribution, and both disorders share common triggers ranging from psychological stress to consuming certain foods [1,5,7]. In a recent cross-sectional study in a combined dermatology-neurology setting, migraine was identified in 54% of patients with rosacea, and rosacea features were present in 65% of patients with migraine, supporting a high degree of bidirectional overlap [5]. These findings are consistent with earlier observations that first highlighted the rosacea-migraine link, particularly in women, and support the concept that these may represent intersecting neurovascular disorders rather than isolated cutaneous or neurologic entities [1,5,8]. 

Several population-based and clinic-based studies have further quantified this comorbidity [5-7]. In a large nationwide cohort, patients with rosacea, particularly women, had a significantly increased risk of incident migraine compared with matched controls, with a reported hazard ratio of 1.24 (95% CI: 1.17-1.32, p < .001) [6]. Other work has shown that individuals with rosacea are more likely to experience migraine-type headaches than controls (18.0% vs. 9.0%, p = 0.011), and that rosacea severity correlates positively with migraine severity (r = 0.284, p < 0.05) [7]. The relationship between rosacea and migraine was initially described predominantly in postmenopausal women, but more recent data indicate that male patients with rosacea may also have up to a twofold increased odds of self-reported migraine, highlighting that sex-related patterns are complex and may vary across populations [8,9].

The mechanistic basis of this association appears to center on shared neurovascular and neuroinflammatory pathways [3,5,7]. Both rosacea and migraine involve activation of trigeminal afferents and dysregulation of cranial and facial vasculature, leading to neurogenic inflammation and abnormal vasodilation [1,3,5]. Trigeminovascular activation promotes the release of vasoactive neuropeptides such as substance P and calcitonin gene-related peptide (CGRP), which contribute to vasodilation, plasma extravasation, and pain signaling in migraine and are increasingly recognized as important mediators in rosacea pathophysiology [3,5,7]. In addition, neurogenic rosacea has emerged as a proposed distinct subtype characterized predominantly by severe flushing, burning pain, dysesthesia, and heightened neurologic comorbidity and is often considered within the erythematotelangiectatic spectrum or as an overlapping phenotype driven by neurovascular dysregulation [1,5]. In neurogenic rosacea, upregulation of CGRP and pituitary adenylate cyclase-activating polypeptide-38 (PACAP‑38) has been proposed to underlie exaggerated facial flushing, burning pain, and sensory hypersensitivity, further supporting a shared neuropeptidergic axis linking skin and meningeal vasculature [5].

These convergent pathways create a rationale for re-examining both diagnosis and management [5-7]. From a diagnostic perspective, recognition of shared triggers, overlapping symptomatology within the trigeminal distribution, and an increased prevalence of migraine in rosacea patients, and vice versa, may prompt more systematic screening for comorbid disease and facilitate earlier identification of high-risk individuals [5-7]. Therapeutically, agents targeting CGRP signaling, originally developed for migraine prevention, are now being explored as potential treatments for refractory flushing and erythema in rosacea, while PACAP-directed therapies under development for headache disorders may also have relevance for cutaneous neurovascular inflammation [5]. Beyond neurologic overlap, rosacea has been linked to inflammatory bowel disease, cardiovascular disease, and other systemic conditions, emphasizing the need to move beyond a purely cutaneous framework when evaluating and managing affected patients [1]. This broader systemic perspective is consistent with emerging neurologic literature suggesting that modifiable biological factors, including vitamin D status, may influence neuroinflammatory disease susceptibility through immunomodulatory, anti-inflammatory, antioxidant, neurotrophic, and blood-brain barrier-preserving mechanisms [10]. In light of these observations, interdisciplinary collaboration between dermatology and neurology is essential to elucidate shared mechanisms, refine clinical phenotyping, and guide mechanism-based treatment strategies [1,3]. The objective of this review is to synthesize current evidence on the neurovascular and neuroinflammatory overlap between rosacea and migraine, highlight existing and emerging therapeutic intersections, and identify key gaps and priorities for future translational and clinical research.

## Review

Methods

Study Design

This study was conducted as a narrative review aimed at synthesizing current evidence on the shared neurovascular and neuroinflammatory mechanisms linking rosacea and migraine, as well as their overlapping clinical features and therapeutic implications.

Literature Search Strategy

A structured literature search was performed using PubMed/Medical Literature Analysis and Retrieval System Online (MEDLINE), Scopus, and Google Scholar to identify relevant studies published up to 2025.

Search terms included combinations of keywords and Medical Subject Headings (MeSH) such as “rosacea” AND "migraine"; “neurogenic inflammation” AND "rosacea"; “trigeminovascular system”; “calcitonin gene-related peptide (CGRP)” AND “rosacea” OR "migraine"; “PACAP” AND “migraine” OR "rosacea"; and “TRP channels” OR “TRPV1” OR “TRPA1” AND “rosacea” OR “migraine.”

Inclusion and Exclusion Criteria

Studies were included if they investigated rosacea, migraine, or their overlap; explored pathophysiological mechanisms (e.g., neuropeptides, TRP channels, trigeminal pathways); examined clinical features, triggers, or therapeutic approaches; were peer-reviewed articles, including original research, clinical trials, systematic reviews, and meta-analyses; and were published in English.

Studies were excluded if they were non-peer-reviewed sources (e.g., opinion pieces without supporting data); lacked relevance to neurovascular or neuroinflammatory mechanisms; or focused solely on unrelated dermatologic or neurologic conditions.

 *Data Selection and Synthesis*

Relevant studies were screened based on title and abstract, followed by a full-text review for eligibility. Data were extracted and synthesized qualitatively, with emphasis on shared neurovascular and neuroinflammatory pathways; common environmental and dietary triggers; overlapping clinical features and sensory phenomena; emerging therapeutic intersections, particularly CGRP- and PACAP-targeted therapies. Given the narrative design, no formal risk-of-bias assessment or quantitative meta-analysis was performed. Instead, priority was given to high-quality and recent evidence, with particular emphasis on mechanistic and translational studies.

Clinical features and triggers

Rosacea most commonly presents with recurrent or persistent centrofacial erythema, episodic flushing, telangiectasias, papules, and pustules. In some patients, phymatous change and ocular involvement are typically localized within the ophthalmic and maxillary branches of the trigeminal nerve (V1-V2) [1,2,11]. Many patients also report burning, stinging, or dysesthetic facial sensations, which may be exaggerated in so-called neurogenic rosacea and can be disproportionate to visible inflammatory lesions [11,12]. Migraine, in contrast, is characterized by recurrent attacks of moderate-to-severe headache that are typically unilateral, pulsating, and aggravated by routine activity, often accompanied by nausea, photophobia, phonophobia, and sometimes aura phenomena, such as visual or sensory disturbances [3,4]. Clinically, migraine pain may involve orbital/periorbital, frontal, or temporal regions, although these locations are not part of the formal International Classification of Headache Disorders, 3rd Edition (ICHD-3) diagnostic criteria [13]. Although the primary symptom complexes differ, cutaneous vascular and sensory symptoms in rosacea versus deep head pain and systemic features in migraine, both disorders preferentially involve trigeminal territories and follow a relapsing-remitting course strongly modulated by environmental and internal triggers [14,15].

Overlapping environmental and dietary triggers

Patients with rosacea frequently identify heat (hot weather, hot baths, saunas), ultraviolet exposure, hot beverages, spicy foods, alcohol, particularly red wine, and emotional stress as triggers of flushing and erythema [12,16]. Dietary studies and patient surveys consistently implicate capsaicin‑containing foods (chili peppers), cinnamaldehyde‑containing foods (tomatoes, citrus, chocolate, cinnamon), and alcohol as among the most common precipitants of rosacea flares, although individual sensitivity is heterogeneous [17]. Similarly, large observational series in migraine identify stress, sleep disturbance, hormonal fluctuations, alcohol, and specific foods (e.g., aged cheeses, processed meats, chocolate, red wine) as frequent attack triggers in susceptible individuals [18]. A comparative summary of these shared trigger classes, underlying molecular pathways, clinical manifestations, and candidate biomarkers is provided in Table 1.

**Table 1:** Shared neurovascular trigger pathways in rosacea and migraine (molecular mechanisms, clinical manifestations, and biomarker correlates) This table summarizes shared trigger classes in rosacea and migraine, linking environmental and physiological stimuli to common neurovascular pathways, including TRPV1/TRPV4 and TRPA1 activation, Ca²⁺ influx, and downstream CGRP/SP-mediated neurogenic inflammation and vasodilation [10-13,17-19]. Clinically, these mechanisms manifest as flushing, warmth, burning, and erythema in rosacea, and as trigeminovascular activation, meningeal vasodilation, and attack precipitation in migraine, with stress-related hypothalamic and autonomic modulation also contributing [1,3,4,10,11,13,17,19]. Candidate biomarkers and readouts reflecting these pathways include plasma CGRP and histamine levels, TRP channel expression, provocation testing (e.g., capsaicin, alcohol), thermography, and functional or physiological measures such as heart rate variability and neuroimaging of hypothalamic and trigeminal structures [3,4,12,14-19]. TRP: transient receptor potential; SP: substance P; CGRP: calcitonin gene-related peptide

Trigger class	Key molecular pathways	Clinical expression in rosacea	Clinical expression in migraine	Candidate biomarkers/readouts
Heat / hot beverages	TRPV1/TRPV4 activation on sensory nerves and keratinocytes → Ca²⁺ influx → SP, CGRP release → neurogenic vasodilation.	Sudden facial flushing, warmth, burning, increased erythema; sometimes worse papules/pustules.	Precipitation of attacks in susceptible patients via hypothalamic and trigeminovascular activation.	Plasma CGRP, facial thermography, TRPV1/TRPV4 expression in skin biopsies.
Spicy foods (capsaicin)	Direct TRPV1 agonism → neuropeptide (CGRP, SP) release; mast cell degranulation and histamine release.	Rapid-onset flushing, warmth, burning or stinging, especially in neurogenic rosacea.	Reported dietary trigger via trigeminal activation and meningeal vasodilation.	Plasma CGRP, capsaicin provocation tests, and neuropeptide levels in skin microdialysate.
Cinnamaldehyde foods (tomato, citrus, chocolate, cinnamon)	TRPA1 activation on C‑fibers and keratinocytes → neurogenic inflammation, ROS‑mediated pathways, PAR2 cross‑talk.	Episodic flushing, erythema, and burning, typically in a centrofacial distribution.	Less consistently reported; may contribute via trigeminal sensitization and vascular reactivity.	TRPA1 expression, ROS and cytokine profiles, and challenge‑linked symptom diaries.
Alcohol (especially red wine)	Ethanol, histamine, tyramine → vasodilation; TRPV1 activation → CGRP release; mast cell activation.	Prominent flushing, warmth, erythema; sometimes edema.	Common trigger (20-50%); immediate and delayed attacks via meningeal vasodilation and CGRP release.	Plasma CGRP and histamine, standardized alcohol challenge, symptom scoring.
Emotional stress	Hypothalamic-autonomic activation; HPA axis changes; modulation of trigeminal and parasympathetic tone.	Exacerbation of flushing, burning, and erythema during stress or embarrassment.	One of the most frequent triggers associated with central sensitization and poly‑trigger patterns.	Heart rate variability, stress hormone profiles, and functional imaging of hypothalamic and trigeminal nuclei.

These overlapping triggers suggest shared upstream pathways involving thermoregulation, autonomic tone, mast cell activation, and trigeminovascular excitability, even though the primary clinical expression, visible facial flushing versus headache, is different [3,19]. Recognition of such common triggers is clinically useful, as it allows clinicians to counsel patients with either disorder (or both) about coordinated trigger avoidance and to consider overlapping mechanisms when evaluating refractory symptoms.

Trigger-induced TRP channel activation and neuropeptide release

Transient receptor potential (TRP) channels, particularly TRPV1, TRPA1, and TRPV4, act as molecular sensors for thermal, chemical, dietary, and inflammatory stimuli and provide a mechanistic link between common rosacea and migraine triggers. In rosacea, TRP channels are expressed on cutaneous sensory nerves, keratinocytes, endothelial cells, and immune cells. Heat, capsaicin, ethanol, and acidic conditions primarily activate or sensitize TRPV1, whereas cinnamaldehyde-containing foods activate TRPA1. These pathways permit calcium influx and stimulate the release of vasoactive neuropeptides, including CGRP and substance P (SP). This cascade promotes neurogenic vasodilation, plasma extravasation, mast cell degranulation, histamine release, cathelicidin production, and inflammatory cell recruitment, clinically manifesting as flushing, burning, erythema, and trigger-induced flares [12,14]. Upregulated TRPV4 and other TRP isoforms may further amplify rosacea flares by promoting SP release, mast cell degranulation, histamine release, cathelicidin production, and downstream vascular inflammation [11,17,18]. Ethanol may further amplify this response by lowering the activation threshold of TRPV1, helping explain why small amounts of alcohol can provoke prominent flushing in susceptible patients [14].

A parallel process occurs in migraine, although the dominant anatomic target is the trigeminovascular system rather than the superficial facial microvasculature. TRPV1 and TRPA1 are expressed on trigeminal afferents innervating meningeal vessels, where they may be activated by endogenous lipids, protons, reactive oxygen species, environmental irritants, alcohol, and other dietary, environmental, or inflammatory triggers. Their activation promotes CGRP release, meningeal vasodilation, and increased trigeminal neuronal excitability, contributing to peripheral and central sensitization during migraine attacks [18-20]. PACAP-38 adds another layer to this shared neuropeptidergic model: it can experimentally induce migraine-like attacks and, in the same provocation models, produce facial flushing and edema resembling rosacea flares [21,22]. Together, these findings support a model in which environmental, dietary, and stress-related triggers activate TRP channels and hypothalamic-autonomic circuits, resulting in CGRP-, SP-, and PACAP-mediated neurogenic inflammation in both facial skin and meninges, with disease-specific clinical expression [19-23].

Recent experimental work further connects TRP activation to downstream immune dysregulation. Capsaicin-responsive, high-sensitivity sensory neurons can worsen rosacea-like dermatitis by releasing CGRP, which then acts on γδ T cells to drive interleukin-17A production [24]. This suggests that TRP channel activation may not only produce acute neurovascular symptoms but also amplify inflammatory pathways relevant to rosacea chronicity. Because TRPV1 and TRPA1 are co-expressed on trigeminal and cutaneous nociceptors, therapeutic strategies that modulate TRP channel sensitization, while preserving physiological temperature sensing, may eventually complement CGRP- or PACAP-directed approaches in patients with overlapping rosacea and migraine [25].

Differences in clinical expression

Despite shared triggers and overlapping neurovascular pathways, rosacea and migraine differ in their dominant clinical manifestations. In rosacea, repeated neurovascular activation manifests primarily as visible centrofacial flushing, persistent erythema, telangiectasias, papulopustules, and, in some patients, tissue hypertrophy and phymatous change, often accompanied by ocular surface disease [10,11]. Neurogenic rosacea is characterized by prominent burning and stinging, dysesthesia, and warmth that may be disproportionate to the degree of visible inflammation, along with marked trigger sensitivity and impaired quality of life [1,10,17].

By contrast, migraine presents as deep, throbbing head pain with systemic autonomic symptoms (nausea, vomiting), sensory hypersensitivity (photophobia, phonophobia, osmophobia), and sometimes neurologic aura, typically without overt cutaneous vascular signs aside from occasional facial pallor or flushing during attacks [3,4,14]. Thus, while both disorders reflect trigeminal and autonomic dysregulation, rosacea primarily externalizes as a cutaneous neurovascular phenotype, whereas migraine primarily manifests as a central pain disorder involving the meninges and brainstem nociceptive circuits [10,17]. Appreciating this distinction is crucial to avoid conflation while still recognizing shared pathophysiology.

Shared sensory hypersensitivity

Sensory hypersensitivity represents an important point of convergence. Many patients with rosacea describe burning, stinging, itching, and exaggerated responses to minor thermal or mechanical stimuli, features that align with the broader concept of “sensitive skin” [13,17]. Neurogenic rosacea in particular has been proposed to represent a small facial fiber neuropathy, with evidence of reduced intraepidermal nerve fiber density, abnormal quantitative sensory testing, and neuropathic‑type pain symptoms in some patients [24].

Migraine is likewise associated with cutaneous allodynia, pain evoked by normally non‑painful stimuli on the scalp or face, which reflects central sensitization of trigeminal pathways [20]. Experimental provocation with CGRP or PACAP‑38 in migraineurs can reproduce not only headache but also facial warmth and flushing, underscoring a shared neuropeptidergic basis for sensory dysregulation across skin and meninges [20-22]. These observations suggest that aberrant processing of thermal and mechanical stimuli in trigeminal small fibers and central pathways contributes both to facial dysesthesia in rosacea and to allodynia in migraine, particularly in patients who exhibit combined disease phenotypes [24].

Potential biomarkers for predicting comorbidity

Several emerging biomarkers may help identify patients at heightened risk for combined rosacea-migraine or for severe neurovascular phenotypes, although none are yet validated for routine clinical use. Cross‑sectional case-control data demonstrate that plasma CGRP levels are significantly higher in individuals with rosacea than in healthy controls, independent of age, sex, body mass index, rosacea subtype, or comorbid migraine, implicating systemic CGRP dysregulation in rosacea pathophysiology [15,20]. More granular work in neurogenic rosacea shows that serum CGRP levels are further elevated in this subtype and correlate with flushing severity and overall disease activity, suggesting a role for CGRP as an adjunct biomarker to identify neurogenic phenotypes and monitor treatment response [17,23].

In migraine, interictal and ictal CGRP concentrations correlate with attack occurrence and may stratify patients more likely to respond to CGRP-targeted therapies, raising the possibility that shared CGRP signatures could mark individuals prone to both migraine and rosacea [4,20]. PACAP‑38 hypersensitivity may represent another cross‑cutting biomarker: intravenous PACAP‑38 reliably induces migraine‑like headaches in a high proportion of migraine and post‑traumatic headache patients and can simultaneously trigger rosacea‑like flushing and edema that are attenuated by sumatriptan in experimental models [22].

Beyond circulating neuropeptides, structural and functional measures of small fiber integrity and sensory processing, such as intraepidermal nerve fiber density on facial skin biopsy, quantitative sensory testing of thermal and mechanical thresholds, and potentially neuroimaging markers of trigeminal and hypothalamic activation, have been proposed as candidate biomarkers for neurogenic rosacea and may help identify patients at particular risk of coexistent migraine [24]. Upregulated expression of TRPV1/TRPA1/TRPV4, increased mast cell density, and elevated proinflammatory cytokines in rosacea skin represent additional tissue‑level signatures that may serve as mechanistic biomarkers, although their predictive value for migraine comorbidity remains speculative [19,24]. Prospective, biomarker‑driven cohort studies specifically designed to assess rosacea‑migraine overlap will be necessary to determine the clinical utility of these candidates.

Pathophysiology

Neurogenic Inflammation and the Trigeminal System

Neurogenic inflammation occurs when activation of peripheral sensory neurons leads to antidromic release of vasoactive neuropeptides, producing vasodilation, increased vascular permeability, and recruitment of immune cells in the affected tissue [26]. Within the trigeminovascular system, trigeminal afferents innervating meningeal and pial vessels release CGRP, SP, and related mediators, which in turn activate and sensitize second-order neurons in the trigeminal nucleus caudalis, contributing to migraine pain [3,20]. More broadly, systemic modulators of neuroinflammation may influence neurologic disease susceptibility; vitamin D, for example, has been implicated in neuroprotection through immunomodulatory, anti-inflammatory, antioxidant, neurotrophic, and blood-brain-barrier-preserving effects [10,27].

A related process has been proposed in rosacea at the level of facial skin. Trigeminal sensory fibers supplying the dermal microvasculature and adnexal structures release CGRP, SP, and other neuropeptides, contributing to flushing, persistent erythema, and burning or stinging reported by many patients, especially those with the neurogenic subtype [1,17]. Experimental work and translational studies suggest a shared role for the trigeminal ganglion as an integrative hub in both disorders, with co‑expression of CGRP, SP, and PACAP in neurons that project both to meningeal vessels and to facial skin [20,28]. This common neuroanatomical substrate may help explain overlapping symptoms in the trigeminal distribution among patients with comorbid rosacea and migraine.

Meningeal Versus Facial Vasculature

Despite convergent neurogenic mechanisms, migraine and rosacea differ in their principal vascular targets. In migraine, neuropeptide release predominantly affects the meningeal and pial vasculature: CGRP‑ and SP‑mediated vasodilation and plasma extravasation at dural vessels activate nociceptive afferents and propagate signals centrally, culminating in headache [3,20]. In rosacea, the primary targets are superficial cutaneous microvessels of the face, where neuropeptide-driven vasodilation and increased blood flow manifest as visible flushing, persistent erythema, and, over time, vascular remodeling with telangiectasia [1,2].

Clinically, this anatomic divergence explains why neurogenic inflammation in rosacea presents as a predominantly visible vascular and sensory phenotype, whereas in migraine it presents as deep, throbbing head pain with comparatively subtle or transient cutaneous changes [3,17]. Nonetheless, the shared cascade of trigeminal activation, neuropeptide release, and neurovascular inflammation unites the two conditions at a mechanistic level.

Calcitonin Gene‑Related Peptide (CGRP)

CGRP is a 37-amino acid neuropeptide abundantly expressed in small‑diameter trigeminal sensory neurons and is one of the most potent endogenous vasodilators identified to date [20]. During spontaneous or provoked migraine attacks, circulating and jugular CGRP levels rise substantially, and exogenous CGRP infusion can trigger migraine-like headaches in susceptible individuals, establishing a causal role in migraine pathophysiology [3,20]. As discussed previously, clinical biomarker studies also implicate CGRP dysregulation in rosacea and neurogenic rosacea, where it may help identify neurovascular phenotypes with heightened flushing, sensory symptoms, or comorbid migraine risk. 

At the receptor level, CGRP binds a heterodimeric receptor composed of calcitonin receptor-like receptor and receptor activity-modifying protein 1, leading to cyclic AMP formation, protein kinase A activation, mast cell degranulation, and secondary cytokine release [20]. Immunohistochemical findings of increased CGRP-positive dermal nerve fibers in lesional rosacea skin further support a cutaneous role for this pathway [16,17]. Together, these mechanisms provide a potential bridge between trigeminovascular activation in migraine and cutaneous neurogenic inflammation in rosacea. The therapeutic relevance of CGRP signaling is highlighted by the success of CGRP-targeted monoclonal antibodies and receptor antagonists in migraine prevention, as well as preliminary evidence that erenumab may reduce persistent erythema and flushing in refractory rosacea [20,29]. These findings position CGRP as a shared effector molecule and suggest that CGRP inhibition may modulate both peripheral cutaneous inflammation and central trigeminovascular sensitization in patients with combined diseases.

Substance P (SP)

SP is a tachykinin neuropeptide frequently co‑stored with CGRP in peptidergic C‑fibers and acts primarily via the neurokinin‑1 (NK1) receptor, as well as mas‑related G protein‑coupled receptors on mast cells [25,27]. SP promotes vasodilation and markedly increases vascular permeability while also driving mast cell degranulation with the release of histamine, leukotrienes, and pro-inflammatory cytokines, thereby amplifying local inflammation and edema [25,28].

In rosacea, lesional facial skin shows an increased density of SP‑positive nerve fibers around superficial vessels and adnexal units, often in close apposition to mast cells and inflammatory infiltrates and, in many cases, co‑expressing the heat‑sensitive channel TRPV1 [17,24]. In migraine, SP contributes to neurogenic inflammation of the meninges by promoting plasma protein extravasation from dural vessels and sensitizing nociceptors, complementing the vasodilatory and pronociceptive actions of CGRP [20,27]. Experimental studies indicate that SP and CGRP together produce greater vasodilation and vascular leakage than either peptide alone, supporting a synergistic role in sustaining neurogenic inflammation and pain in both facial skin and meningeal tissues [20,25].

Pituitary Adenylate Cyclase‑Activating Polypeptide (PACAP)

PACAP‑38 is a multifunctional neuropeptide with potent vasodilatory and immunomodulatory effects and has emerged as another key mediator linking migraine and rosacea [3,4]. Intravenous PACAP‑38 infusion in migraine patients reliably induces delayed migraine‑like attacks accompanied by cephalic vasodilation, confirming its role in migraine pathogenesis [4,29].

In rosacea, a human provocation model has demonstrated that PACAP‑38 infusion can elicit pronounced facial flushing, erythema, and edema that closely mimic spontaneous flares; these responses are attenuated by sumatriptan, indicating partial overlap with established antimigraine pathways [21]. PACAP acts through PAC1, VPAC1, and VPAC2 receptors to increase intracellular cyclic AMP, relax vascular smooth muscle, and enhance the release of inflammatory mediators from endothelial and immune cells [20,30]. A PACAP‑neutralizing monoclonal antibody (Lu AG09222) has been shown to prevent PACAP‑38-induced migraine attacks and associated vasodilation in humans, highlighting PACAP as a promising therapeutic target and suggesting potential applicability of PACAP‑directed strategies in rosacea as well [29,31].

Gaps in the current literature

Despite growing interest in the neurovascular overlap between rosacea and migraine, current evidence remains largely observational and mechanistic, with limited interventional or biomarker-driven data [5,32]. Clinical evaluation of CGRP-targeted therapy in rosacea is currently restricted to a single-center, open-label trial of erenumab in patients with refractory flushing and persistent erythema [25,33]. Although this study provides preliminary proof of concept, its small sample size, lack of placebo control, and short follow-up limit conclusions regarding efficacy, optimal dosing, long-term safety, and applicability across rosacea endotypes [25].

PACAP-focused evidence is similarly preliminary. A double-blind, placebo-controlled crossover provocation study demonstrated that PACAP-38 infusion can induce rosacea-like flushing, supporting a potential mechanistic role for PACAP in rosacea-associated neurovascular activation [20]. However, this acute provocation model does not establish whether chronic PACAP blockade is effective or safe in rosacea populations, and long-term PACAP-targeted interventional trials have not yet been conducted [28,34,35].

Similarly, therapies that might simultaneously benefit rosacea and migraine have been poorly studied. Existing CGRP‑inhibitor trials enroll patients on the basis of skin disease alone, with no systematic assessment of headache outcomes; migraine trials of CGRP or PACAP inhibitors likewise omit dermatologic phenotyping. Traditional treatments such as beta‑blockers and triptans are used empirically for flushing or provocation models, but there are no controlled dual‑endpoint studies evaluating their efficacy in patients who have both conditions.

Epidemiologic work hints that sex and age modulate the co‑occurrence of rosacea and migraine. Women with rosacea have a higher hazard of migraine, and men have about twice the odds of self‑reported migraine, yet existing cohorts lack power and rarely collect detailed hormonal, reproductive, or age‑related vascular data. Methodological issues are pervasive: most comorbidity studies are cross‑sectional or retrospective with heterogeneous diagnostic criteria, small samples, and modest adjustment for confounders. As a result, the field lacks large, multi-center randomized controlled trials focusing on the neurovascular intersection of rosacea and migraine.

Non‑migraine headaches are another blind spot. Nearly one‑third of rosacea patients report headaches, with a substantial minority experiencing tension‑type or other headaches. However, most studies prioritize migraine phenotyping and lump non‑migraine headaches together or exclude them entirely, leaving it unclear whether mechanisms identified in rosacea generalize to other headache disorders. Finally, although elevated plasma CGRP and other neurogenic markers have been observed in rosacea and migraine, these biomarkers have not been used prospectively to enrich clinical trials, and large cohorts such as Copenhagen Rosacea Cohort (COROCO) and Copenhagen Migraine Cohort (COMICO) remain largely descriptive.

Future directions

Biomarker-Driven Dual-Endpoint Clinical Trials

The most urgent need is for adequately powered, randomized, placebo‑controlled trials that test CGRP‑ and PACAP‑targeted agents in rosacea while measuring both skin and headache outcomes. The open‑label erenumab study provides proof‑of‑concept, and PACAP‑38 provocation models highlight PACAP as a dual target, but definitive efficacy and safety require multi‑center randomized controlled trials (RCTs) with dermatologic and neurologic endpoints in the same participants. These trials should incorporate biomarker stratification, e.g., selecting subgroups with elevated plasma CGRP, PACAP hypersensitivity, or small‑fiber neuropathy, to test whether circulating neuropeptides, TRP channel expression, or nerve‑fiber measures predict response. Objective vascular imaging, quantitative sensory testing, and standardized symptom scores should be embedded in these designs to validate mechanistic hypotheses.

Standardized Phenotyping and Longitudinal Epidemiology

Heterogeneous diagnostic criteria hamper cross‑study synthesis. Adopting consensus‑based definitions for rosacea subtypes (including neurogenic rosacea) and ICHD definitions for migraine and other headaches will allow meaningful comparisons across cohorts. Prospective cohorts such as COROCO and COMICO provide a platform for standardized dermatologic and neurologic assessments; future studies should leverage these resources to collect longitudinal data on age‑by‑phenotype and sex‑by‑phenotype interactions, including hormonal status, reproductive history, and vascular parameters. Broader epidemiological investigations must also classify tension‑type, cluster, and secondary headaches using standardized criteria and relate them to rosacea features and neurogenic markers.

Mechanism-Based Personalization and Novel Biomarker Discovery

Moving toward personalized care will require integrating clinical phenotypes with biomarker signatures. Prospective studies should evaluate whether baseline or dynamic changes in CGRP or PACAP predict the development of migraine in rosacea cohorts or response to targeted therapies. Systematic assessment of small‑fiber neuropathy markers (e.g., intraepidermal nerve fiber density, sensory thresholds) may identify patients who would benefit from neuromodulatory agents. Integrative omics approaches, transcriptomics, proteomics, and metabolomics of skin, blood, and cerebrospinal fluid, combined with high‑resolution vascular imaging, could reveal new neuropeptides, receptors, and ion channels and help construct multidimensional neurovascular endotypes. Embedding such stratification strategies within multi‑center trials and validating them in real‑world cohorts will enable precision therapy selection.

Interdisciplinary Collaboration and Translational Infrastructure

Finally, sustained collaboration between dermatology and neurology is essential. Joint clinics, shared registries and harmonized research protocols would facilitate systematic phenotyping, foster recognition of high‑risk neurovascular endotypes and support integrated management strategies instead of siloed care. Such collaborations are particularly important for designing and executing complex mechanistic and therapeutic studies that require expertise in headache phenotyping, advanced imaging and neuropeptide biology.

**Figure 1:**
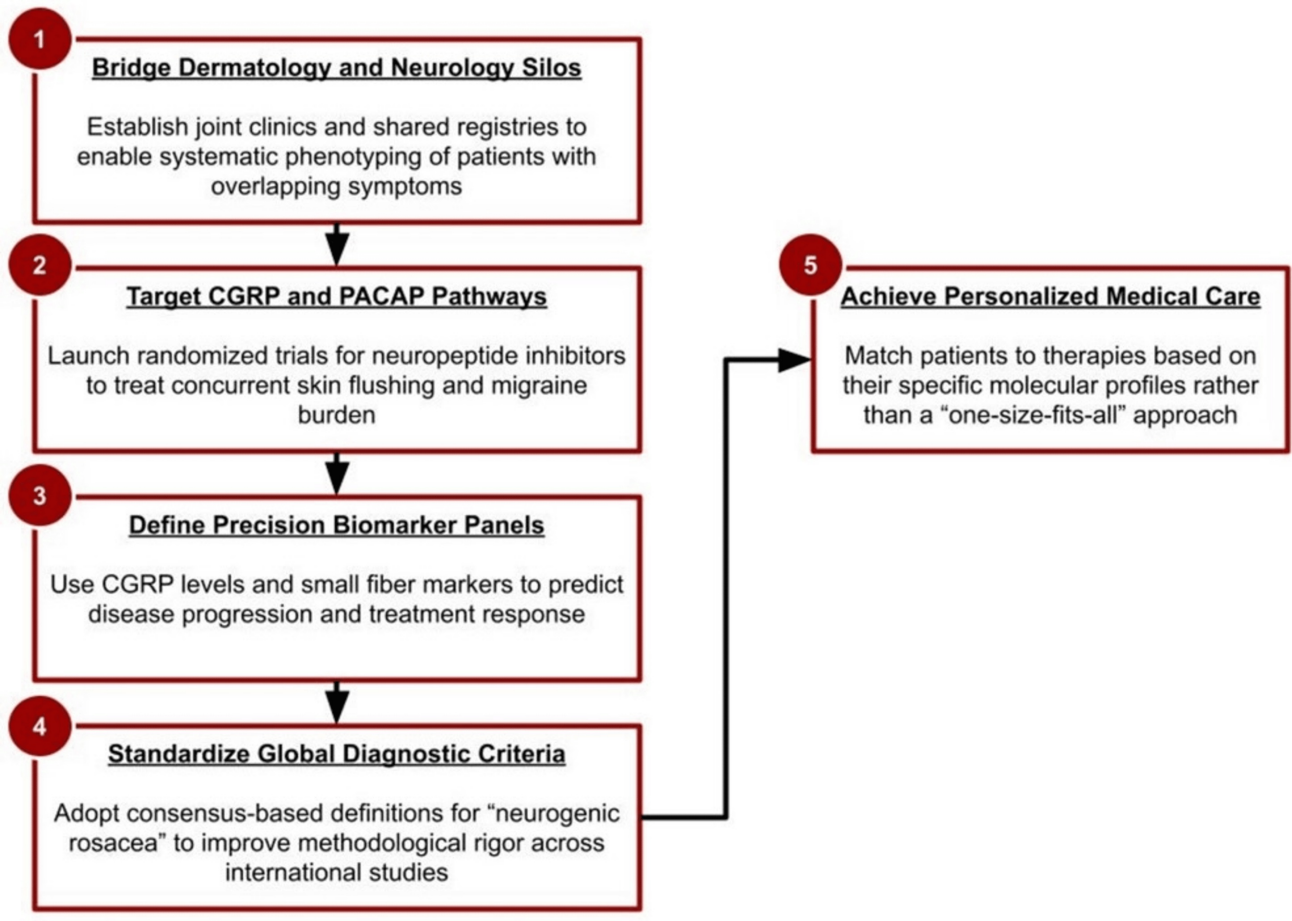
Proposed future directions for mechanism-based research and personalized care in rosacea-migraine overlap This flowchart outlines a potential framework for advancing research and clinical translation in patients with overlapping rosacea and migraine features. Initial priorities include bridging dermatology and neurology silos through joint clinics and shared registries, followed by targeted randomized trials of CGRP- and PACAP-directed therapies. These efforts would support the development of precision biomarker panels, including CGRP levels and small fiber neuropathy markers, while also strengthening diagnostic consistency through standardized criteria for neurogenic rosacea and related headache phenotypes. Together, these advances aim to enable personalized medical care in which patients are matched to therapies based on molecular profiles and clinical endotypes rather than a one-size-fits-all approach. Figure 1 was created by the authors using Google Docs (Google LLC, Mountain View, California, USA). CGRP: calcitonin gene-related peptide; PACAP: pituitary adenylate cyclase-activating polypeptide

A comparative summary of these shared trigger classes, proposed molecular pathways, clinical manifestations, and candidate biomarker readouts is provided in Figure 1.

## Conclusions

Emerging evidence firmly supports the concept that rosacea and migraine are interrelated neurovascular disorders linked by shared neuroinflammatory mechanisms, and this shared biology is reinforced by overlapping environmental and dietary triggers. From a treatment perspective, these shared mechanisms support a growing case for using similar therapeutic strategies. CGRP‑targeted monoclonal antibodies and receptor antagonists have shown early promise in attenuating persistent erythema and flushing in refractory rosacea. While PACAP‑38 provocation models demonstrate that a PACAP blockade may represent another dual‑use target at the skin-brain interface. Off‑label use of beta‑blockers for rosacea‑associated flushing and their established role in migraine prevention further underscore the therapeutic continuity between the two conditions, even as rigorous, dual‑endpoint trials remain scarce. 

Addressing these challenges will require deeper and more systematic collaboration between dermatology and neurology. Interdisciplinary cohorts (e.g., COROCO, COMICO), combined clinics, and shared research infrastructure enable harmonized phenotyping, standardized diagnostics, and coordinated longitudinal follow-up in patients with overlapping rosacea and headache disorders. Such platforms are essential for validating candidate biomarkers, including CGRP, PACAP, small fiber measures, and TRP‑channel signatures across diverse populations and for understanding how sex, age, and hormonal milieu shape neurovascular risk and treatment response over time. Ultimately, the convergence of mechanistic, epidemiologic, and early interventional data points toward a future in which care for patients at the rosacea‑migraine interface is patient‑centered and mechanism‑based rather than organ‑based. By integrating clinical phenotype, trigger profile, comorbidities, and biomarker signatures, it should become possible to stratify patients into neurovascular endotypes that guide targeted biological and neuromodulatory therapies, while minimizing unnecessary exposure to ineffective treatments. Achieving this vision will require well-designed, multi-center, biomarker-driven trials and sustained interdisciplinary collaboration. If realized, it could significantly improve outcomes and quality of life for individuals living with both rosacea and migraine.
